# Case report: Rapid development of acute symptomatic portal vein system thrombosis after endoscopic variceal therapy in a patient with liver cirrhosis

**DOI:** 10.3389/fmed.2024.1382181

**Published:** 2024-04-23

**Authors:** Ran Wang, Xiaozhong Guo, Fangbo Gao, Yongguo Zhang, Qianqian Li, Siqi Jia, Xiaodong Shao, Xingshun Qi

**Affiliations:** Department of Gastroenterology, General Hospital of Northern Theater Command, Shenyang, China

**Keywords:** portal vein system thrombosis, superior mesenteric vein thrombosis, endoscopic variceal treatment, anticoagulation therapy, liver cirrhosis

## Abstract

Acute portal vein thrombosis (PVST), a serious complication of liver cirrhosis, is characterized as abdominal pain secondary to intestinal ischemia, and even intestinal necrosis. Anticoagulation is recommended for the treatment of acute PVST, but is often postponed in cirrhotic patients with acute variceal bleeding or those at a high risk of variceal bleeding. Herein, we reported a 63-year-old male with a 14-year history of alcoholic liver cirrhosis who developed progressive abdominal pain related to acute portal vein and superior mesenteric vein thrombosis immediately after endoscopic variceal ligation combined with endoscopic cyanoacrylate glue injection for acute variceal bleeding. Fortunately, acute PVST was successfully recanalized by the use of low molecular weight heparin. Collectively, this case suggests that acute symptomatic PVST can be secondary to endoscopic variceal therapy in liver cirrhosis, and can be safely and successfully treated by anticoagulation.

## 1 Introduction

Portal vein system thrombosis (PVST), mainly including portal vein thrombosis (PVT), superior mesenteric vein (SMV) thrombosis, and splenic vein thrombosis, is a common complication of liver cirrhosis (1), with a prevalence of 11.18%–16.91% and an incidence of 8.16%–12.92% (2). While PVST is often asymptomatic in liver cirrhosis and even transient (3), a subset of PVST patients may develop acute and progressive manifestations related to intestinal ischemia, such as abdominal pain, and even intestinal necrosis (4).

The occurrence of PVST is generally multifactorial. Its local risk factors often include splenectomy, splenic arterial embolization, and intra-abdominal surgery (5). But it remains controversial about whether endoscopic variceal therapy (EVT) is a risk factor for PVT among studies. Politoske et al. (6) concluded that the incidence of PVT following EVT was similar to that in unselected patients with cirrhosis and portal hypertension. By comparison, accumulative evidence from our group and others have supported EVT as a potential risk factor for the development of PVST in liver cirrhosis (7, 8).

Anticoagulation is recommended for the treatment of PVST in liver cirrhosis (9), considering its efficacy in achieving portal vein recanalization (10). However, anticoagulation is often postponed or avoided in cirrhotic patients with acute variceal bleeding (AVB) or those at a high risk of bleeding. Until now, the evidence regarding initiation of anticoagulation is still insufficient in such patients.

Herein, we reported a case of liver cirrhosis where acute symptomatic PVST developed soon after EVT, but was successfully recanalized by anticoagulation.

## 2 Case description

**FIGURE 1:**
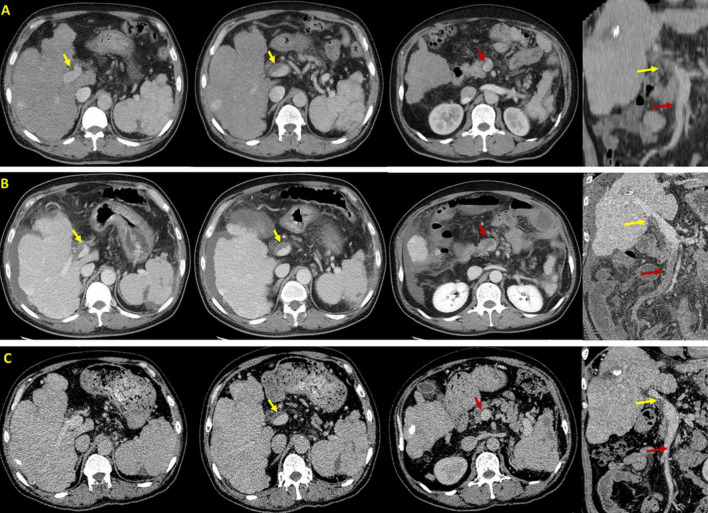
Axial and coronal contrast-enhanced computed tomography scans in this patient. **(A)** Before EVT, CT scans performed on 7 November 2020 demonstrated PVT (yellow arrow) without SMV thrombosis (red arrow). **(B)** After EVT, CT scans performed on 29 November 2020 demonstrated PVT (yellow arrow) with acute SMV thrombosis (red arrow). **(C)** After anticoagulation, CT scans performed on 19 January 2021 demonstrated that PVT remains stable (yellow arrow) and SMV thrombosis disappeared (red arrow).

On 23 November 2020, a 63-year-old male admitted to our department due to hematemesis and melena for a duration of 4 days. Upon admission, the patient did not have abdominal pain, distension, or fever. He was diagnosed with alcoholic liver cirrhosis in 2006. He had repeatedly undergone EVT for gastroesophageal variceal bleeding since 2012. He was also diagnosed with hepatocellular carcinoma (HCC) in 2013 and had been repeatedly treated with transcatheter arterial chemoembolization (TACE) since then. The last TACE procedure was performed on 6 November 2020, and his liver lesion was stable at this admission. He was treated with partial splenic embolization for hypersplenism in 2014. Notably, before this admission, he performed contrast-enhanced computed tomography (CT) scans on 7 November 2020, which revealed only partial thrombosis within the main portal vein (Figure 1A).

On November 27, the patient underwent EVT, including variceal ligation for severe esophageal varices and injection of a mixture of cyanoacrylate and glucose (1 ml 50% glucose + 1 ml cyanoacrylate + 1 ml 50% glucose) for severe gastric varices with adherent clot. At the same day, the patient’s general condition remains stable without abdominal pain or fever. On November 29, he developed acute persistent abdominal pain and mild fever (37.5°C) with an elevated D-dimer level of 17.06 mg/L (Table 1). Emergency abdominal CT angiography (Figure 1B) suggested acute extensive thrombosis extended to the SMV. Considering that active gastrointestinal bleeding stopped with a stable hemoglobin level and esophageal and gastric varices had been treated with EVT, anticoagulation therapy was immediately initiated after the patient and his family members sufficiently understood the risk of anticoagulation and the patient’s wife signed the written informed consents. Subcutaneous injection of low molecular weight heparin (LMWH) with a dosage of 5,000 IU bid was immediately administered. Subsequently, abdominal pain and fever gradually resolved within 2 days. Contrast-enhanced CT scans were repeated on 19 January 2021, showing recanalization of SMV thrombosis and partial thrombosis within the main portal vein (Figure 1C). LMWH was maintained for 6 months and then switched to oral rivaroxaban. The patient remained asymptomatic without recurrent thrombosis until melena recurred on 4 August 2023. He underwent tissue adhesive injection for gastric varices at our department. Oral rivaroxaban was re-titrated after EVT. At the last follow-up visit on 14 December 2023, he remained stable without rebleeding events.

**TABLE 1:** Laboratory tests of this patient.

Laboratory examination	Results			Unit	Normal range
	At admission (23/11/2020)	After EVT (29/11/2020)	After anticoagulation (6/12/2020)		
PT	14.8	**16.6**	**15.6**	s	11.5–14.5
INR	1.22	1.41	1.3	/	/
APTT	42.2	44.3	56.8	s	28.0–40.0
FIB	2.36	2.43	2.25	g/L	2.00–4.00
D-dimer	1.56	**17.06**	**3.81**	mg/L FEU	0.01–0.55
PLT	145	166	186	× 10^9^/L	125–350
WBC	4.3	**15.6**	4.5	× 10^9^/L	3.5–9.5
NEU	3.29	**13.2**	3.08	× 10^9^/L	1.8–6.3
RBC	3.77	4.38	3.95	× 10^12^/L	4.3–5.8
Hb	84	98	95	g/L	130–175
TBIL	14.6	22.3	16	μmol/L	5.1–22.2
ALT	7.3	5.27	4.92	U/L	9–50
AST	13.12	13.04	16.55	U/L	15–40
AKP	104.96	95.22	77.82	U/L	45–125
GGT	50.03	45.33	50.47	U/L	10–60
ALB	30.9	34.5	31.1	g/L	40–55
LIPA	**359**	41	NA	U/L	23–300
AMY	43	30	NA	U/L	30–110
CRP	11.04	**90.14**	**44.61**	mg/L	≤10

EVT, endoscopic variceal therapy; PT, prothrombin time; INR, international normalized ratio; APTT, activated partial thromboplastin time; FIB, fibrinogen; PLT, platelet; WBC, white blood cell; NEU, neutrophils; RBC, red blood cell; Hb, hemoglobin; TBIL, total bilirubin; ALT, alanine aminotransferase; AST, aspartate aminotransferase; AKP, alkaline phosphatase; GGT, gamma-glutamyl transpeptidase; ALB, albumin; LIPA, lipase; AMY, amylase; CRP, C-reactive protein; NA, not available. The bold values indicate significantly abnormal changes in the laboratory tests.

## 3 Discussion

### 3.1 PVST after EVT

The pathogenesis of thrombus formation should be classical Virchow’s triad: decreased blood flow velocity, vascular endothelial injury, and hypercoagulable state (11). In liver cirrhosis, increased portal pressure can reduce blood flow velocity, which may be a main risk factor of PVST development (12). Similarly, there is an increased incidence of PVT with deterioration of liver function (13). In our case, it can be proposed that EVT induces an alteration of portal hemodynamics, leading to a transient increase in portal pressure and an increased risk of PVT. However, the correlation between EVT and PVT is still debated among literature (6–8). Our case had been diagnosed with partial PVT which was asymptomatic, but experienced acute symptomatic PVST extending to the SMV within only 2 days after EVT. Furthermore, he had imaging evidence before and after EVT, which clearly indicates its impact on acute thrombus extension. Recent evidence also supports a causal effect estimation of EVT, particularly endoscopic sclerotherapy (7), variceal ligation, and variceal ligation combined with endoscopic cyanoacrylate glue injection (8), with the risk of PVST. The underlying mechanism of PVST formation after EVT needs to be further explored. Regardless, the risk of PVST should be closely screened in patients undergoing EVT.

Certainly, our case also involves other possible factors contributing to thrombus formation, including prior history of HCC, TACE, and splenic arterial embolization (5). However, they might be associated with partial PVT before this admission, rather than acute SMV thrombosis at this admission.

**FIGURE 2:**
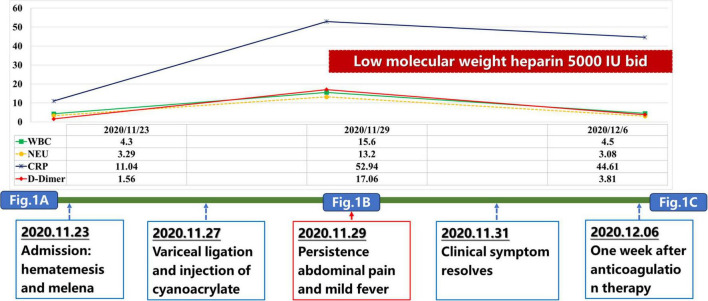
A time-line of this patient’s disease course.

Our case also had elevated WBC and C-reactive protein (CRP) levels after EVT, which was in parallel with the development of acute PVT event (Figure 2). This phenomenon suggested that thrombo-inflammation should be the potential mechanism of PVT in our case. Liver cirrhosis is associated with activation of systemic inflammation, which may increase the risk of PVST (14, 15). It has been shown that inflammatory markers, such as CRP, tumor necrosis factor α, procalcitonin, and interleukin 6, were positively correlated with PVT in cirrhotic patients (16, 17). In turn, thrombosis also exacerbates inflammation mediated by endothelial cells, leukocytes, and platelets (18). PVT can also directly occlude the portal vein lumen, slowing blood flow and increasing the chance of intestinal bacterial translocation, thereby raising the risk of systemic inflammation (19). Despite the role of local inflammation as a precipitating factor of thrombus formation has been well known (16), our case did not present with any inflammation-related manifestations before this acute PVST event. Instead, fever occurred after a diagnosis of PVST, indicating that local intestinal blood stasis should induce inflammation reaction. After anticoagulation, the patient’s temperature gradually normalized without anti-inflammatory medications. Collectively, inflammation should be concomitant with acute PVST event, but may not be a risk factor for acute PVST.

Cancer itself can also cause the hypercoagulability in this patient, leading to a higher incidence of PVT (20). In patients with HCC, the development of PVT might be associated with various causes, including direct tumor compression, tumor secondary hypoxia, and circulating tumor cells (21). Furthermore, tumors can increase inflammatory factors, such as tumor necrosis factor α, posing this patient at a hypercoagulable state (20, 22).

Impact of D-dimer on the development of PVT should not be neglected in this case. D-dimer level was slightly elevated upon admission, but significantly increased after EVT (Figure 2). The change of D-dimer level correlates positively with the progression of PVT. This is consistent with the findings of our previous meta-analysis that D-dimer serves as a predictive marker for PVT in cirrhosis, and postoperative D-dimer, rather than preoperative D-dimer, should be of significance on the development of PVT (23). This emphasizes the importance of monitoring D-dimer levels after EVT or surgery for earlier detection of PVT.

Another possibility should not be ignored that endoscopic cyanoacrylate injection caused glue migration, thereby inducing the development of PVST (24). Cyanoacrylate glue induced PVST, which is well-known, but rare, is reported in 2% patients who underwent endoscopic cyanoacrylate injection for gastric varices (25). But this possibility cannot be supported, because the SMV patency was immediately achieved following anticoagulation therapy in our case.

### 3.2 Anticoagulation for PVST

Anticoagulation is recommended as the first-line treatment for acute symptomatic PVST (9). According to the data from a meta-analysis, the pooled rate of overall bleeding, upper gastrointestinal bleeding, and major bleeding in patients with cirrhosis and PVST after anticoagulation was 10.3%, 3.2%, and 2.8%, respectively (7). Concerns about the risk of bleeding associated with anticoagulation often limit its use in patients with AVB. Notably, in our case, the onset of acute PVST followed a recent AVB event. Despite so, anticoagulation had to be immediately administered for acute symptomatic extensive PVST presenting with persistent abdominal pain secondary to intestinal ischemia to maximize the rate of portal vein recanalization. Finally, our case achieved a resolution of clinical symptoms and recanalization of SMV thrombosis after anticoagulation without any bleeding event. Thus, our case further confirms that once hemostasis is achieved, early anticoagulation is safe and effective in cirrhosis. This is consistent with the findings from a recent multi-centric randomized controlled trial by Gao et al. and a recent meta-analysis by our group (26, 27).

Thrombolytic therapy is an alternative choice of treatment for acute symptomatic PVT (28). As previously reported, it should be more effective (29, 30). However, it carries a potentially higher risk of bleeding (28). Therefore, it was not considered as the first-line choice in our current case with AVB following EVT. Transjugular intrahepatic portosystemic shunt should also be another choice in our case, if anticoagulation fails or is not feasible (31, 32).

According to the current practice guidelines for the diagnosis and treatment of PVT and deep vein thrombosis, the recommended dosage of LMWH should be 100 U/kg twice daily (9, 33). Our patient weighed approximately 60 kg. Thus, theoretically, LMWH should be given at a dosage of 6,000 U subcutaneous injection every 12 h. However, our patient received LMWH at a dosage of 5000 U subcutaneous injection every 12 h. This was attributed to two considerations. First, he had recently experienced AVB before anticoagulation, raising concerns about the risk of rebleeding secondary to a therapeutic dosage of anticoagulation. Hence, we preferred to reduce the dosage of LMWH slightly. Second, the specific dosage of LMWH per syringe is 5,000 U at our hospital. Thus, it is also more convenient for our clinical practice, as compared to 6,000 U.

### 3.3 Follow-up

During the follow-up period, our case received long-term anticoagulation therapy without any adjustment of other regimens. Notably, there was a notable decrease in the frequency of bleeding events, including 11 AVB episodes within 8 years before anticoagulation, but only one re-bleeding episode 3 years after anticoagulation. This phenomenon is consistent with the findings of our meta-analysis that anticoagulation may decrease the incidence of variceal bleeding in cirrhotic patients with PVST to some extent (7). We speculate that this benefit results from the improvement of microvascular thrombosis and decrease of portal vein pressure after long-term anticoagulation therapy.

## 4 Conclusion

Our case emphasizes the necessity of screening for PVST after EVT in cirrhotic patients, and also supports the efficacy and safety of anticoagulation for PVST in the case of high-risk bleeding. Further cohort studies are very necessary to validate this conclusion.

## Data availability statement

The original contributions presented in this study are included in this article/supplementary material, further inquiries can be directed to the corresponding authors.

## Ethics statement

Ethical approval was not required as this is a single case report and does not include identifiable data of the patient. The studies were conducted in accordance with the local legislation and institutional requirements. The participants provided their written informed consent to participate in this study. Written informed consent was obtained from the individual(s) for the publication of any potentially identifiable images or data included in this article.

## Author contributions

RW: Writing – original draft, Writing – review & editing. XG: Writing – review & editing. FG: Writing – review & editing. YZ: Writing – review & editing. QL: Writing – review & editing. SJ: Writing – review & editing. XS: Writing – review & editing. XQ: Writing – original draft, Writing – review & editing, Conceptualization, Supervision.
